# 3,4,5-Trihy­droxy­benzohydrazide

**DOI:** 10.1107/S1600536811034374

**Published:** 2011-08-27

**Authors:** Uzma Ashiq, Rifat Ara Jamal, Sammer Yousuf

**Affiliations:** a Department of Chemistry, University of Karachi, Karachi-75270, Pakistan; b HEJ Research Institute of Chemistry, International Center for Chemical and Biological Sciences, University of Karachi, Karachi-75270, Pakistan

## Abstract

In the title compound, C_7_H_8_N_2_O_4_, the dihedral angle between the aromatic ring and the hydrazide grouping is 21.34 (7)°. In the crystal, the mol­ecules are linked into a three-dimensional network by O—H⋯O, O—H⋯N and N—H⋯O hydrogen bonds.

## Related literature

For the biological activity of hydrazides, see: Maqsood *et al.* (2006 ▶). For related structures, see: Jamal *et al.* (2009 ▶); Saeed *et al.* (2008 ▶); Zareef *et al.* (2006 ▶).
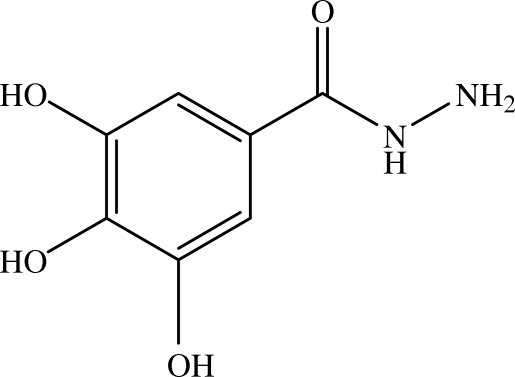


## Experimental

### 

#### Crystal data

C_7_H_8_N_2_O_4_
*M* *_r_* = 184.15
Monoclinic, 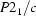

*a* = 3.7307 (3) Å
*b* = 22.8402 (18) Å
*c* = 8.7064 (7) Å
β = 93.290 (2)°
*V* = 740.65 (10) Å^3^
*Z* = 4
Mo *K*α radiation
μ = 0.14 mm^−1^
*T* = 273 K
0.28 × 0.21 × 0.20 mm

#### Data collection

Bruker SMART APEX CCD area-detector diffractometer
Absorption correction: multi-scan (*SADABS*; Bruker, 2000 ▶) *T*_min_ = 0.963, *T*_max_ = 0.973
4345 measured reflections
1352 independent reflections
1234 reflections with *I* > 2σ(*I*)
*R*_int_ = 0.016

#### Refinement

*R*[*F* ^2^ > 2σ(*F* ^2^)] = 0.034
*wR*(*F* ^2^) = 0.092
*S* = 1.09
1352 reflections
150 parameters
H atoms treated by a mixture of independent and constrained refinement
Δρ_max_ = 0.18 e Å^−3^
Δρ_min_ = −0.24 e Å^−3^

### 

Data collection: *SMART* (Bruker, 2000 ▶); cell refinement: *SAINT* (Bruker, 2000 ▶); data reduction: *SAINT*; program(s) used to solve structure: *SHELXS97* (Sheldrick, 2008 ▶); program(s) used to refine structure: *SHELXL97* (Sheldrick, 2008 ▶); molecular graphics: *SHELXTL* (Sheldrick, 2008 ▶); software used to prepare material for publication: *SHELXTL*, *PARST* (Nardelli, 1995 ▶) and *PLATON* (Spek, 2009 ▶).

## Supplementary Material

Crystal structure: contains datablock(s) global, I. DOI: 10.1107/S1600536811034374/hb6363sup1.cif

Structure factors: contains datablock(s) I. DOI: 10.1107/S1600536811034374/hb6363Isup2.hkl

Supplementary material file. DOI: 10.1107/S1600536811034374/hb6363Isup3.cml

Additional supplementary materials:  crystallographic information; 3D view; checkCIF report

## Figures and Tables

**Table 1:** Hydrogen-bond geometry (Å, °)

*D*—H⋯*A*	*D*—H	H⋯*A*	*D*⋯*A*	*D*—H⋯*A*
O1—H1*A*⋯O2^i^	0.85 (3)	2.09 (2)	2.8254 (14)	145.0 (19)
N1—H1*B*⋯O1^ii^	0.86 (2)	2.24 (2)	2.9960 (15)	146.2 (16)
O2—H2*A*⋯N2^iii^	0.91 (3)	1.80 (2)	2.6877 (17)	165 (2)
N2—H2*B*⋯O3^iv^	0.88 (2)	2.25 (2)	3.1158 (17)	167.0 (18)
N2—H2*C*⋯O4^v^	0.92 (2)	2.454 (18)	3.2255 (18)	141.8 (15)
O3—H3*A*⋯O4^vi^	0.89 (2)	1.77 (2)	2.6522 (15)	171 (2)

Symmetry codes: (i) 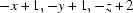; (ii) 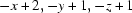; (iii) 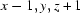; (iv) 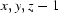; (v) 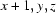; (vi) 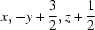.

**Fig. 1.**
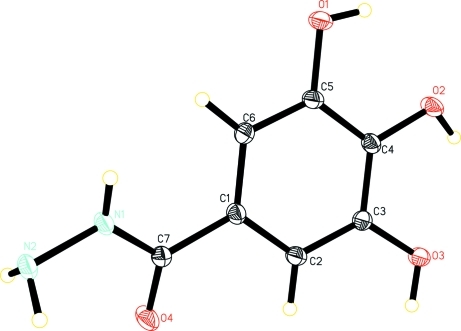
The molecular structure of (I) with displacement ellipsoids drawn at 50% probability level.

**Fig. 2.**
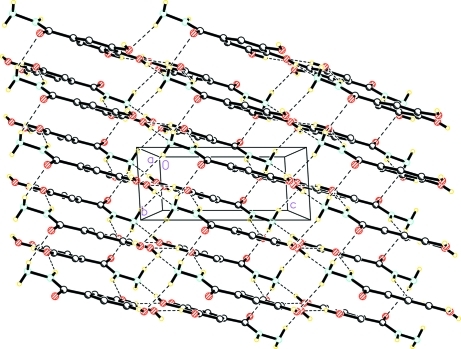
The crystal packing of the title compound I. Only hydrogen atoms involved in hydrogen bonding are shown.

## supplementary crystallographic information

### Comment

In order to further explore the biological significance of hydrazides, we have prepared the title compound (I).  It  was found to be active against DPPH radical scavenging activity and inactive against all fungal strains (Maqsood *et al.* 2006). The crystal structures of trimethoxybenzohydrazide (Saeed *et al.* 2008, Zareef *et al.* 2006) and *para* hydroxybenzohydrazide (Jamal *et al.* 2009) analogues of (I) have already been reported.

The molecular structure of (I) is composed of a hydrazide moiety attached to the phenyl ring (Fig. 1). The phenyl ring is almost planar with a maximum deviation of 0.009 (1) Å from the least-squares plane. The bond lengths and angles all are in normal range as in other structurally related compounds (Saeed *et al.* 2008; Zareef *et al.*, 2006). In the crystal, the molecules are linked to form three-dimensional molecular network *via* O1—H1A···O2, N1—H1B···O1, O2—H2A···N2, N2—H2B···O3, N2—H2C···O4 and O3—H3A···O4 intermolecular hydrogen bonds (Tab. 1 & Fig. 2).

### Experimental

To a solution of methyl-3,4,5-trihydroxybenzoate (3.68 g, 20 mmol) in 75 ml ethanol, hydrazine hydrate (5.0 ml, 100 mmol) was added. The mixture was refluxed for 5 h and a solid was obtained upon removal of the solvent by rotary evaporation. The resulting solid was washed with hexane to afford 3,4,5-trihydroxybenzohydrazide (yield 87%) (Maqsood *et al.*, 2006). Colourless blocks of (I) were grown from a solution of methanol by slow evaporation at room temperature.

### Refinement

The H atoms on the N atoms (N–H= 0.92 (2)–0.86 (19) Å) O atoms (O–H= 0.91 (3)–0.85 (19) Å) and Carbon (C–H= 0.961 (18)–0.955 (17) Å) atoms were located in difference Fourier maps and refined isotropically.

### Crystal data

**Table:**

C_7_H_8_N_2_O_4_	*F*(000) = 384
*M**_r_* = 184.15	*D*_x_ = 1.651 Mg m^−^^3^
Monoclinic, *P*2_1_/*c*	Mo *K*α radiation, λ = 0.71073 Å
Hall symbol: -P 2ybc	Cell parameters from 2464 reflections
*a* = 3.7307 (3) Å	θ = 3.0–28.3°
*b* = 22.8402 (18) Å	µ = 0.14 mm^−^^1^
*c* = 8.7064 (7) Å	*T* = 273 K
β = 93.290 (2)°	Block, colorles
*V* = 740.65 (10) Å^3^	0.28 × 0.21 × 0.20 mm
*Z* = 4	

### Data collection

**Table:**

Bruker SMART APEX CCD area-detector diffractometer	1352 independent reflections
Radiation source: fine-focus sealed tube	1234 reflections with *I* > 2σ(*I*)
graphite	*R*_int_ = 0.016
ω scan	θ_max_ = 25.5°, θ_min_ = 1.8°
Absorption correction: multi-scan (*SADABS*; Bruker, 2000)	*h* = −4→4
*T*_min_ = 0.963, *T*_max_ = 0.973	*k* = −27→26
4345 measured reflections	*l* = −10→10

### Refinement

**Table:**

Refinement on *F*^2^	Primary atom site location: structure-invariant direct methods
Least-squares matrix: full	Secondary atom site location: difference Fourier map
*R*[*F*^2^ > 2σ(*F*^2^)] = 0.034	Hydrogen site location: inferred from neighbouring sites
*wR*(*F*^2^) = 0.092	H atoms treated by a mixture of independent and constrained refinement
*S* = 1.09	*w* = 1/[σ^2^(*F*_o_^2^) + (0.0509*P*)^2^ + 0.2069*P*] where *P* = (*F*_o_^2^ + 2*F*_c_^2^)/3
1352 reflections	(Δ/σ)_max_ = 0.001
150 parameters	Δρ_max_ = 0.18 e Å^−^^3^
0 restraints	Δρ_min_ = −0.24 e Å^−^^3^

### Special details

**Table:**

Geometry. All e.s.d.'s (except the e.s.d. in the dihedral angle between two l.s. planes) are estimated using the full covariance matrix. The cell e.s.d.'s are taken into account individually in the estimation of e.s.d.'s in distances, angles and torsion angles; correlations between e.s.d.'s in cell parameters are only used when they are defined by crystal symmetry. An approximate (isotropic) treatment of cell e.s.d.'s is used for estimating e.s.d.'s involving l.s. planes.
Refinement. Refinement of *F*^2^ against ALL reflections. The weighted *R*-factor *wR* and goodness of fit *S* are based on *F*^2^, conventional *R*-factors *R* are based on *F*, with *F* set to zero for negative *F*^2^. The threshold expression of *F*^2^ > σ(*F*^2^) is used only for calculating *R*-factors(gt) *etc*. and is not relevant to the choice of reflections for refinement. *R*-factors based on *F*^2^ are statistically about twice as large as those based on *F*, and *R*- factors based on ALL data will be even larger.

### Fractional atomic coordinates and isotropic or equivalent isotropic displacement parameters (Å^2^)

**Table:**

	*x*	*y*	*z*	*U*_iso_*/*U*_eq_	
O1	0.6088 (3)	0.49395 (4)	0.75022 (12)	0.0388 (3)	
O2	0.4790 (3)	0.57014 (4)	0.97692 (11)	0.0328 (3)	
H2A	0.375 (6)	0.5994 (11)	1.029 (3)	0.069 (7)*	
O3	0.5451 (3)	0.68880 (5)	0.93130 (11)	0.0334 (3)	
H3A	0.602 (6)	0.7255 (10)	0.907 (2)	0.061 (6)*	
O4	0.7876 (3)	0.70585 (5)	0.36218 (12)	0.0402 (3)	
N1	1.0268 (3)	0.62028 (5)	0.30646 (13)	0.0270 (3)	
H1B	1.100 (5)	0.5854 (9)	0.329 (2)	0.037 (5)*	
N2	1.1408 (4)	0.64212 (6)	0.16535 (13)	0.0283 (3)	
H2C	1.285 (5)	0.6739 (9)	0.188 (2)	0.041 (5)*	
H2B	0.950 (6)	0.6553 (8)	0.112 (2)	0.044 (5)*	
C1	0.7698 (4)	0.63145 (6)	0.55422 (14)	0.0224 (3)	
C2	0.7029 (4)	0.67156 (6)	0.66914 (15)	0.0250 (3)	
H2	0.718 (4)	0.7128 (8)	0.6488 (18)	0.031 (4)*	
C3	0.6093 (4)	0.65203 (6)	0.81195 (14)	0.0235 (3)	
C4	0.5743 (4)	0.59242 (6)	0.84021 (14)	0.0231 (3)	
C5	0.6437 (4)	0.55257 (6)	0.72421 (15)	0.0244 (3)	
C6	0.7421 (4)	0.57157 (6)	0.58183 (15)	0.0246 (3)	
H6	0.780 (4)	0.5428 (7)	0.5049 (19)	0.030 (4)*	
C7	0.8618 (4)	0.65546 (6)	0.40269 (14)	0.0239 (3)	
H1A	0.548 (6)	0.4893 (9)	0.842 (3)	0.057 (6)*	

### Atomic displacement parameters (Å^2^)

**Table:**

	*U*^11^	*U*^22^	*U*^33^	*U*^12^	*U*^13^	*U*^23^
O1	0.0733 (9)	0.0179 (5)	0.0271 (6)	0.0005 (5)	0.0199 (5)	0.0016 (4)
O2	0.0557 (7)	0.0214 (5)	0.0230 (5)	0.0030 (5)	0.0173 (5)	0.0034 (4)
O3	0.0602 (8)	0.0190 (5)	0.0227 (5)	−0.0028 (5)	0.0169 (5)	−0.0024 (4)
O4	0.0707 (9)	0.0224 (5)	0.0295 (6)	0.0123 (5)	0.0199 (5)	0.0066 (4)
N1	0.0409 (8)	0.0210 (6)	0.0202 (6)	0.0058 (5)	0.0118 (5)	0.0038 (4)
N2	0.0388 (8)	0.0276 (7)	0.0194 (6)	0.0021 (6)	0.0105 (5)	0.0028 (5)
C1	0.0257 (7)	0.0229 (7)	0.0190 (6)	0.0014 (5)	0.0045 (5)	0.0011 (5)
C2	0.0332 (8)	0.0188 (7)	0.0235 (7)	−0.0002 (6)	0.0063 (5)	0.0012 (5)
C3	0.0291 (8)	0.0211 (7)	0.0209 (6)	0.0002 (5)	0.0061 (5)	−0.0016 (5)
C4	0.0280 (8)	0.0225 (7)	0.0194 (6)	0.0006 (5)	0.0062 (5)	0.0023 (5)
C5	0.0317 (8)	0.0177 (7)	0.0241 (7)	0.0009 (5)	0.0055 (5)	0.0014 (5)
C6	0.0322 (8)	0.0218 (7)	0.0205 (7)	0.0027 (6)	0.0064 (5)	−0.0023 (5)
C7	0.0302 (8)	0.0211 (7)	0.0207 (6)	0.0001 (5)	0.0047 (5)	0.0000 (5)

### Geometric parameters (Å, °)

**Table:**

O1—C5	1.3654 (16)	N2—H2B	0.88 (2)
O1—H1A	0.85 (2)	C1—C2	1.3897 (18)
O2—C4	1.3603 (16)	C1—C6	1.3934 (19)
O2—H2A	0.91 (3)	C1—C7	1.4869 (17)
O3—C3	1.3678 (16)	C2—C3	1.3844 (18)
O3—H3A	0.89 (2)	C2—H2	0.961 (18)
O4—C7	1.2306 (17)	C3—C4	1.3912 (19)
N1—C7	1.3363 (18)	C4—C5	1.3946 (19)
N1—N2	1.4139 (15)	C5—C6	1.3828 (19)
N1—H1B	0.860 (19)	C6—H6	0.955 (17)
N2—H2C	0.92 (2)		
			
C5—O1—H1A	108.1 (14)	O3—C3—C2	123.28 (12)
C4—O2—H2A	107.7 (15)	O3—C3—C4	116.38 (11)
C3—O3—H3A	110.1 (14)	C2—C3—C4	120.35 (12)
C7—N1—N2	120.34 (12)	O2—C4—C3	123.55 (12)
C7—N1—H1B	124.6 (12)	O2—C4—C5	117.28 (12)
N2—N1—H1B	114.4 (12)	C3—C4—C5	119.17 (12)
N1—N2—H2C	107.3 (11)	O1—C5—C6	119.29 (12)
N1—N2—H2B	107.8 (12)	O1—C5—C4	119.76 (12)
H2C—N2—H2B	106.7 (17)	C6—C5—C4	120.95 (12)
C2—C1—C6	120.31 (12)	C5—C6—C1	119.26 (12)
C2—C1—C7	117.11 (12)	C5—C6—H6	118.0 (10)
C6—C1—C7	122.56 (12)	C1—C6—H6	122.7 (10)
C3—C2—C1	119.94 (13)	O4—C7—N1	119.12 (12)
C3—C2—H2	120.1 (9)	O4—C7—C1	122.66 (12)
C1—C2—H2	119.9 (9)	N1—C7—C1	118.21 (12)
			
C6—C1—C2—C3	0.2 (2)	C3—C4—C5—C6	−0.8 (2)
C7—C1—C2—C3	178.80 (13)	O1—C5—C6—C1	178.56 (13)
C1—C2—C3—O3	178.95 (13)	C4—C5—C6—C1	−0.4 (2)
C1—C2—C3—C4	−1.4 (2)	C2—C1—C6—C5	0.7 (2)
O3—C3—C4—O2	0.5 (2)	C7—C1—C6—C5	−177.85 (13)
C2—C3—C4—O2	−179.16 (13)	N2—N1—C7—O4	5.1 (2)
O3—C3—C4—C5	−178.65 (13)	N2—N1—C7—C1	−175.85 (13)
C2—C3—C4—C5	1.7 (2)	C2—C1—C7—O4	−20.3 (2)
O2—C4—C5—O1	1.0 (2)	C6—C1—C7—O4	158.29 (15)
C3—C4—C5—O1	−179.71 (13)	C2—C1—C7—N1	160.76 (13)
O2—C4—C5—C6	179.99 (13)	C6—C1—C7—N1	−20.7 (2)

### Hydrogen-bond geometry (Å, °)

**Table:**

*D*—H···*A*	*D*—H	H···*A*	*D*···*A*	*D*—H···*A*
O1—H1A···O2^i^	0.85 (3)	2.09 (2)	2.8254 (14)	145.0 (19)
N1—H1B···O1^ii^	0.86 (2)	2.24 (2)	2.9960 (15)	146.2 (16)
O2—H2A···N2^iii^	0.91 (3)	1.80 (2)	2.6877 (17)	165 (2)
N2—H2B···O3^iv^	0.88 (2)	2.25 (2)	3.1158 (17)	167.0 (18)
N2—H2C···O4^v^	0.92 (2)	2.454 (18)	3.2255 (18)	141.8 (15)
O3—H3A···O4^vi^	0.89 (2)	1.77 (2)	2.6522 (15)	171 (2)

Symmetry codes: (i) −*x*+1, −*y*+1, −*z*+2; (ii) −*x*+2, −*y*+1, −*z*+1; (iii) *x*−1, *y*, *z*+1; (iv) *x*, *y*, *z*−1; (v) *x*+1, *y*, *z*; (vi) *x*, −*y*+3/2, *z*+1/2.

## References

[bb1] Bruker (2000). *SADABS*, *SMART* and *SAINT* Bruker AXS Inc., Madison, Wisconsin, USA.

[bb2] Jamal, R. A., Ashiq, U., Arshad, M. N., Maqsood, Z. T. & Khan, I. U. (2009). *Acta Cryst.* E**65**, o1764. 10.1107/S1600536809025094 PMC2977245 21583474

[bb3] Maqsood, Z. T., Khan, K. M., Ashiq, U., Ara, R., Chohan, Z. H., Mahroof-Tahir, M. & Supuran, C. T. (2006). *J. Enzyme Inhib. Med. Chem.* **21**, 37–42. 10.1080/14756360500277459 16570503

[bb4] Nardelli, M. (1995). *J. Appl. Cryst.* **28**, 659.

[bb5] Saeed, A., Mumtaz, A., Rafique, H., Gotoh, K. & Ishida, H. (2008). *Acta Cryst.* E**64**, o2336. 10.1107/S1600536808036301 PMC2959913 21581311

[bb6] Sheldrick, G. M. (2008). *Acta Cryst.* A**64**, 112–122. 10.1107/S0108767307043930 18156677

[bb7] Spek, A. L. (2009). *Acta Cryst.* D**65**, 148–155. 10.1107/S090744490804362X PMC2631630 19171970

[bb8] Zareef, M., Iqbal, R., Qadeer, G., Arfan, M. & Lu, X.-M. (2006). *Acta Cryst.* E**62**, o3259–o3261.

